# Growing Polymer Vesicles Generated by Polymerization Induced Self-Assembly Coupled With a Living Chemical Reactor

**DOI:** 10.3389/fbioe.2020.01018

**Published:** 2020-09-01

**Authors:** Zhihui Lu, Jinshan Guo

**Affiliations:** ^1^Department of Histology and Embryology, School of Basic Medical Sciences, Southern Medical University, Guangzhou, China; ^2^Department of Earth and Planetary Science and Origin of Life Initiative, Harvard University, Cambridge, MA, United States

**Keywords:** chemical oscillation, PISA, polymeric vesicles, self-assembly, biomimetics

## Abstract

Chemical oscillatory reactions have attracted intensive attention due to their autonomous, continuous, and periodic features. Herein, the radicals generated in Belousov-Zhabotinsky (BZ) oscillator was used to initiate RAFT (reversible addition-fragmentation chain transfer) polymerization of 2-methoxyethyl acrylate (MEA) extending from hydrophilic poly(ethylene glycol) chain transfer agent (PEG-CTA) to give amphiphilic block copolymer, which self-assembled into collective objects with a size ranging from sub-micron to micron. Small-to-giant polymer vesicles could be generated using the above-mentioned BZ-PISA technology, the encapsulation of active BZ recipe into the vesicles also endorses the vesicles with growing features with potential for drug delivery and biomedical applications.

Chemical oscillatory reactions have been developed to mimic the behaviors of biochemical oscillations ubiquitously existing in natural living systems, characterizing as periodic, autonomous, and continuous (Kurin-Csörgei et al., 2005; Horváth et al., 2009). Among them, the BZ reaction, firstly developed as the non-organic analog to the Krebs cycle in 1951, is the mostly studied chemical oscillators in non-linear and systems chemistry (Horváth et al., 2009; Nogueira et al., 2014; Ruiz-Mirazo et al., 2014). And due to its stable, long-lasting, and repeatable oscillation features, BZ reaction has also been employed in the development of chemical tuning machine and chemical analog of noisy “metabolism” system, to handle information (Srivastava et al., 2018). The combination of BZ reaction with soft matters or polymeric systems has also led to numerous applications, such as the generation of self-beating or self-oscillating gels, colloidosomes, vesicles, and polymer brushes as well as chemomechanical coupling systems (Pereira de Souza and Perez-Mercader, 2014; Tamate et al., 2014, 2016; Masuda et al., 2015, 2016, 2018; Ren and Perez-Mercader, 2018; Onoda et al., 2017; Hu and Pérez-Mercader, 2018).

Recent years, BZ reaction has been introduced into artificial cell models development, the autonomous self-oscillating feature of BZ reaction allows the oscillating BZ reaction encapsulated liposomes, self-assembled from amphiphilic lipid, to function independently from its surroundings (Tomasi et al., 2014). And in terms of artificial cell models, polymersomes, self-assembled from amphiphilic copolymers, has attract more attentions due to that polymersomes possess higher amphiphilic molecular weights and broader chemical choice over that of lipids (Ren and Perez-Mercader, 2018; Rideau et al., 2018; Guo et al., 2019). The fact that polymerization need radicals and BZ reaction produce radicals further connect BZ reaction and polymersomes together, especially in the context of BZ reaction coupled polymerization induced self-assembly (BZ-PISA) (Bastakoti and Perez-Mercader, 2017a,b; Bastakoti et al., 2018). In BZ-PISA, the radicals produced in BZ reaction are used to drive polymerization of hydrophobic monomer extending from hydrophilic macromolecular RAFT agent to give amphiphilic block copolymers, which concurrently self-assembled into micelles and finally transformed into giant vesicles along with the packing parameter change in the course of the ongoing polymerization (Israelachvili, 2011; Tomasi et al., 2014; Rideau et al., 2018; Cheng and Pérez-Mercader, 2019; Guo et al., 2019). Comparing with conventional PISA that always gives nano-sized vesicles, BZ-PISA leads to giant vesicles benefiting from the autonomous BZ reaction and the hypersaline environment. The encapsulation of an active oscillatory chemical reaction within the polymer vesicles also endow them with important extra functionalities, including the information handling ability mentioned above, because BZ reaction can be used in chemical communication (Pérez-Mercader et al., 2017), and as a chemical turning machine (Albertsen et al., 2017). However, detailed investigation of the polymerization and self-assembly process in BZ-PISA using hydrophilic monomer that polymerized into hydrophobic block has never been documented.

Herein, the radicals generated in BZ reaction is used to drive the polymerization of 2-methoxyethyl acrylate (MEA), a water soluble monomer that becomes hydrophobic after polymerization, extending from hydrophilic poly(ethylene glycol) chain transfer agent (PEG-CTA) to give amphiphilic deblock copolymer, poly(ethylene glycol)-block-poly(2-methoxyethyl acrylate) (PEG-b-PMEA) (Figure 1). The polymerization process of PEG-b-PMEA in BZ-PISA with different target degree of polymerizations (DP_target_s) for MEA of 200, 400, and 600 are studied in detail. And the concurrent self-assembly process the morphologies of different samples at different time is also well investigated.

**FIGURE 1:**
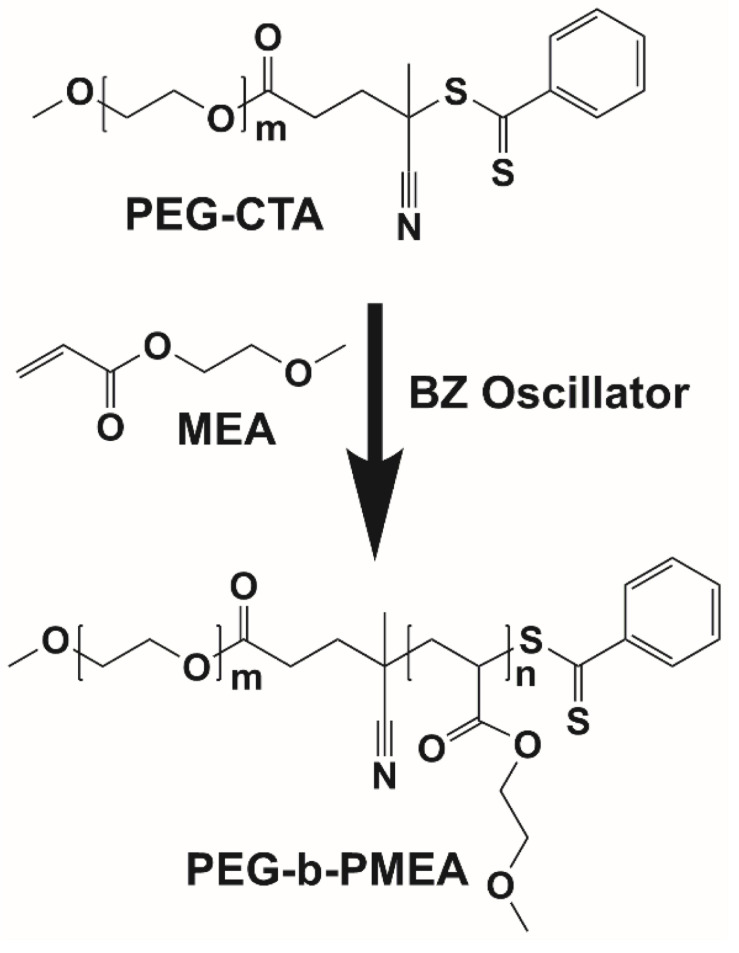
Synthesis of PEG-b-PMEA copolymer via BZ-PISA.

In pure BZ oscillatory reaction (Supplementary Figure S1), the Ru catalyst oscillating between Ru^2+^ and Ru^3+^, resulting in the redox potential oscillation in between ∼860 and ∼1050 mV, with an average period of ∼100 s and an average oscillation amplitude of ∼160 mV. In the BZ-PISA experiments, the mixed solution of PEG-CTA and MEA monomer are fed to the BZ solution, and the reaction mixture is stirred at 200 rpm (Figure 1). Foil film protection is applied to avoid any visible light-induced oxidation/reduction of Ru catalyst, which is also a radical formation process that was used to generate giant vesicles using photo PISA (Albertsen et al., 2017; Ren and Perez-Mercader, 2018). In the case of BZ-PISA for PEG-b-PMEA (DP_target_ = 200), there is a long induction time of ∼70 min (Figure 2A), implying that the consumption of radicals by polymerization temporarily suspended some critical steps in the oscillation cycle documented in previous literature (Bastakoti and Perez-Mercader, 2017a,b), thus affected the oscillatory regime of BZ reaction. After that, the oscillation is restored, with an average period of 102 s and a smaller amplitude of 132 mV. For BZ-PISA of PEG-b-PMA with a DP_target_ of 200, the monomer conversion increased with reaction time increase, the final monomer conversion at 120 min was determined to be ∼90% (Figure 2B). Similarly, the final monomer conversions at 120 min for BZ-PISA of PEG-b-PMEA_400_ and PEG-b-PMEA_600_ were all higher than 90% (Figure 3). The oscillation of BZ-PISA for PEG-b-PMEA exhibited different features compared with the oscillation in pure BZ. As shown in Figure 2C (lower panel), in the oscillatory region, the minimum potential of each oscillation period kept increasing from ∼690 to ∼775 mV. While, in pure BZ oscillatory reaction (Figure 3), the minimum potential of each period kept decreasing from ∼1000 to ∼875 mV (Figure 2C, topper panel). In the cases of BZ-PISA using other monomers, including butyl acrylate (BA) (Bastakoti and Perez-Mercader, 2017a), acrylonitrile (Bastakoti and Perez-Mercader, 2017b), and ethyl acrylate (Bastakoti et al., 2018), the minimum potential of each period also exhibited deceasing trends. This unique feature for BZ-PISA of PEG-b-PMEA might relate to the special chemical structure of MEA, which contains an ethylene glycol unit and an ether group on the side chain, very similar chemical structure with that of PEG. The ^1^H-NMR spectra of the purified PEG-b-PMEA polymers with different DP_target_s and PEG, PEG-CTA are shown in Figure 2D. With the PEG peak (a in Figure 2D) being kept nearly the same, the integration areas from the peaks of PMEA (b to f in Figure 2D, the ^1^H-NMR spectrum of MEA monomer is presented in Supplementary Figure S2 for comparison) became bigger and bigger in the spectra of PEG-PMEA_200_, PEG-PMEA_400_, to PEG-PMEA_600_, indicating the successful introduction of more MEA units into the block copolymers when the feeding ratios of MEA to PEG increased. For BZ-PISA of PEG-b-PMEA_600_, along with reaction time increasing, the increase of the areas of PMEA peaks in the ^1^H-NMR spectra can be clearly observed in Figure 4. These results indicate the decent controllability of BZ-PISA in terms of polymerization and the mutual effect between polymerization and oscillation, which was not detailed documented in previous literatures.

**FIGURE 2:**
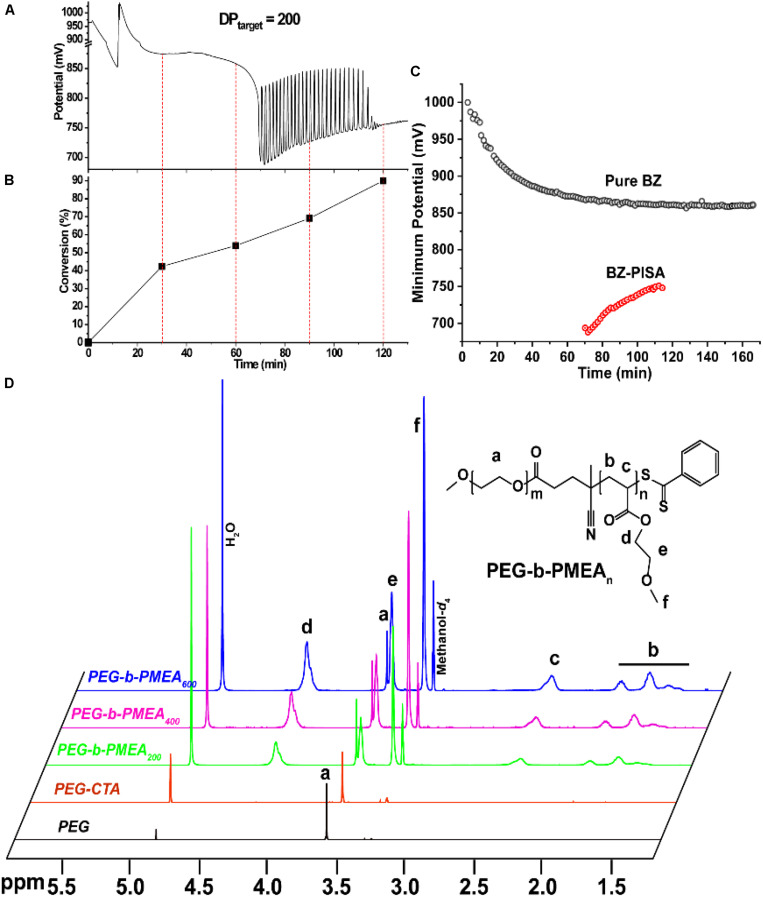
Redox potential oscillation curve **(A)** and monomer conversions **(B)** in the course of BZ-PISA for PEG-b-PMEA with a DP_target_ of 200. **(C)** Change of the minimum potentials of every periods for pure BZ reaction and BZ-PISA of PEG-b-PMA with a DP_target_ of 200. **(D)** ^1^H-NMR spectra of PEG, PEG-CTA and the purified final products of BZ-PISA for PEG-b-PMEA (DP_target_ = 200, 400, and 600) after 2 h reaction.

**FIGURE 3:**
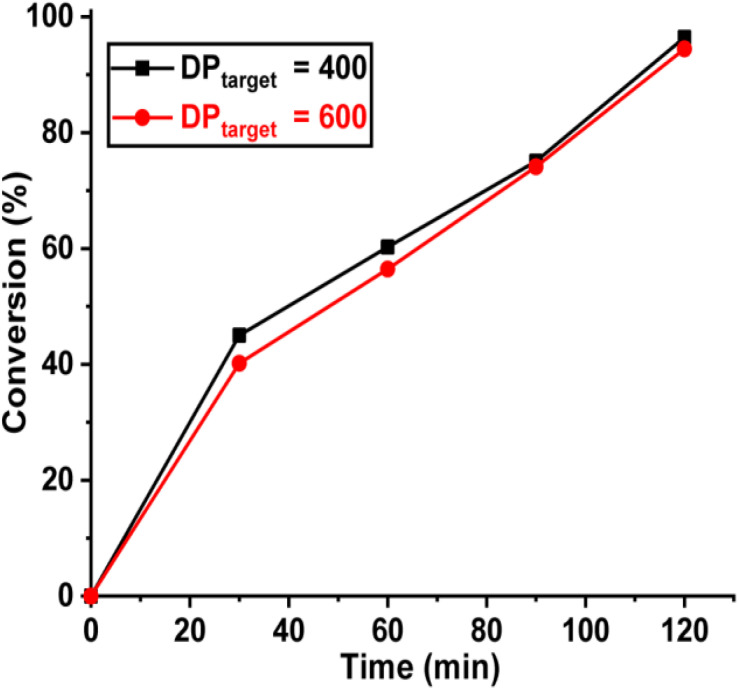
Monomer conversions of BZ-PISA for PEG-b-PMEA with a DP_target_ of 400 and 600 at different time points.

**FIGURE 4:**
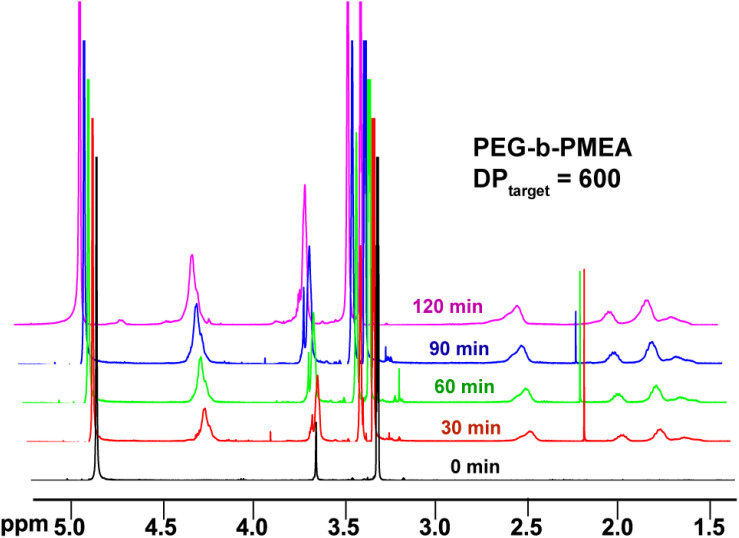
^1^H-NMR spectra of BZ-PISA samples for PEG-b-PMEA (DP_target_ = 600) withdrawn at different time points.

The self-assembly of the obtained amphiphilic block copolymers concurrent with BZ-initiated RAFT polymerization was studied by DLS and TEM measurements (Figure 5). To reflect the real situation and eliminate the reaction time during sample transport, the DLS tests were conduct on site of BZ-PISA reaction. Since MEA is water soluble, just after mixing MEA monomer with BZ recipe, the solution was totally transparent. Along with the dispersion radical polymerization, the solution became more and more turbid. As shown in Figure 5A1, For BZ-PISA of PEG-b-PMEA with a DP_target_ = 200, the hydrodynamic diameters of the self-assembled structures increased from 267 nm at 30 min to 491 nm at 120 min. Vesicular structures are observed from the TEM images of the 60-min sample, and vesicle budding and division can be clearly seen (Figure 5A2 and Supplementary Figure S3), which is further proved by the show up of ellipse and pear-shaped structures in the enlarged TEM images (Supplementary Figure S3). For BZ-PISA of PEG-b-PMEA with a DP_target_ = 400, similar hydrodynamic diameter increase but at a larger scale, from 520 nm at 30 min to 904 nm at 120 min, was also observed (Figure 5B1), and bigger vesicles than that of DP_target_ = 200 sample at 60 min were obtained after 60 min reaction (Figure 5B2). While, when DP_target_ = 600, the hydrodynamic diameters of the self-assembled structures kept decreasing from 3.19 μm at 30 min to 2.01 μm at 120 min (Figure 5C1). In our experiment, the molar amount of PEG-CTA was kept unchanged, increasing of DP_target_ means the increase of monomer content in aqueous solution. At higher monomer content, in the beginning of BZ-PISA reaction, MEA molecules might exist as droplets which were stabilized by the synthesized amphiphilic PEG-b-PMEA polymers, or the monomers were encapsulated in the inner cavity of the self-assembled polymersomes. Thus along with the consumption of monomer by polymerization, the sizes of the self-assembled structures kept decreasing. Polymer vesicles with bigger diameters were observed for the BZ-PISA of PEG-b-PMEA_600_ sample at 60 min (Figure 5C2).

**FIGURE 5:**
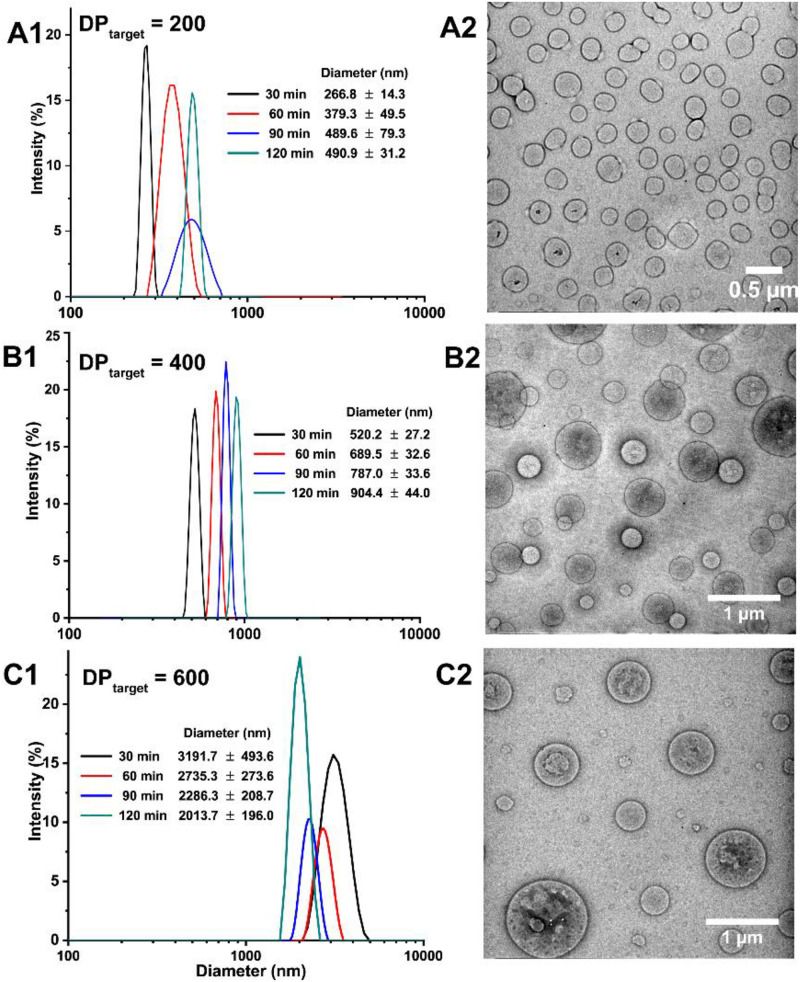
Hydrodynamic diameters of the self-assembled structures of BZ-PISA for PEG-b-PMEA at different time points *in situ* monitored by DLS and corresponding TEM images of the 60-min samples: **(A1,A2)** DP_target_ = 200; **(B1,B2)** DP_target_ = 400; **(C1,C2)** DP_target_ = 600.

To further confirm the generation of giant vesicles through one-pot BZ-PISA, cryo-SEM study on the final product at 120 min of the PEG-b-PMEA with a DP_target_ = 600 was conducted, the results are shown in Figure 6. Cryo-SEM can reflect the real state of the self-assembled structures by freezing the aqueous sample quickly in frozen liquid nitrogen (pretreated under vacuum) to eliminate the formation of ice crystals, and with the help of freeze-fracture, the cross sections and possibly inner features of the self-assembled structures could be captured. From Figure 6, freeze-fractured hollow structures with a diameter ∼ 2 μm could be observed, the cross sections of the vesicle membranes can also be clearly seen. Deformed and collapsed vesicle membranes, which can be clearly seen in Figures 6C,D, reflect the soft feature of the polymersomes. The thickness of vesicle membrane is measured to be ∼30 nm (Figure 6D), which is in the range of 5–50 nm, the reported thickness of the bilayer of polymersomes (Rideau et al., 2018). The cryo-SEM results further confirm the formation of polymer vesicles in BZ-PISA of PEG-b-PMEA.

**FIGURE 6:**
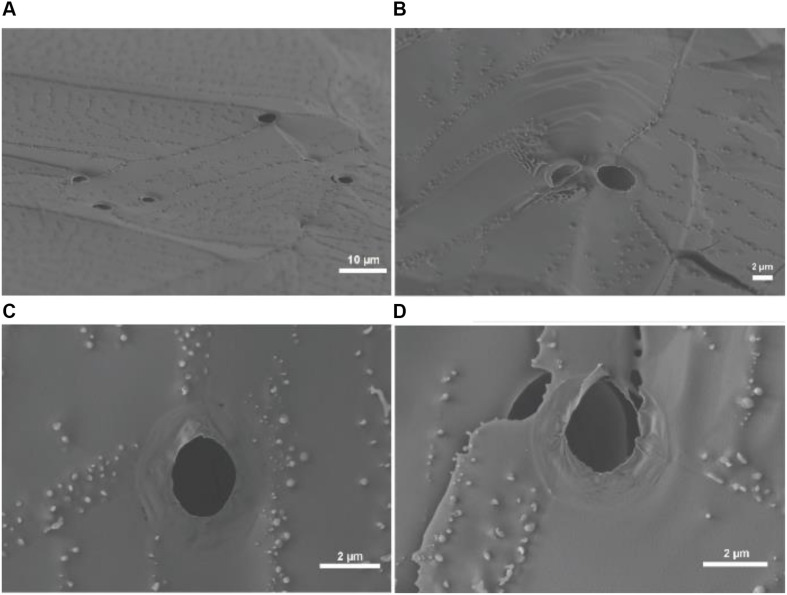
Cryo-SEM images of the self-assembled structures of BZ-PISA for PEG-b-PMEA (DP_target_ = 600) sampled at 120 min.

In conclusion, using the radicals generated in Belousov-Zhabotinsky (BZ) oscillator as initiator, a water soluble monomer, MEA, which became hydrophobic after polymerization, was polymerized extending from the active end of PEG-CTA via RAFT polymerization. The obtained amphiphilic block copolymer was concurrently self-assembled into sub-micron to micron sized collective objects. By adjusting the feeding ratio of MEA monomer to PEG-CTA from 200, 400, to 600, through one-pot BZ-mediated polymerization induced self-assembly (BZ-PISA), polymer vesicles with sizes ranging from 100 nm to ∼2.0 μm were autonomously generated from simple non-amphiphilic building blocks. The encapsulation of active BZ recipe into polymeric giant vesicles endorses them with additional functionalities, including information handling capability, brings closer to the development of functional microreactor as artificial living system.

## Experimental Part

### Materials

Poly(ethylene glycol) 4-cyano-4(phenylcarbonothioylthio) pentanoate (PEG-CTA) was synthesized according to the literature, via the esterification reaction between methoxy poly(ethylene glycol) (mPEG, molecular weight = 1900 Da, Fluka), 4-cyano-4-(thiobenzoylthio) pentanoic acid (CTA, Strem) with the help of N,N′-dicylohexylcarbodiimide (DCC, Sigma-Aldrich) and N,N′-dimethylamino -pyridine (DMAP, Alfa Aesar) (Szymanski and Perez-Mercader, 2016). Methanol-*d*_4_ for ^1^H-NMR tests, was purchased from Cambridge Isotope Laboratories, Inc., 2-Methoxyethyl acrylate (MEA, Sigma-Aldrich), sodium hydroxide (NaOH, Sigma-Aldrich), sodium bromate (NaBrO_3_, Sigma-Aldrich), malonic acid (MA, Sigma-Aldrich), tris(2,2′-bipyridyl) dichlororuthenium(II) hexahydrate (Ru(bpy)_2_Cl_3_, Sigma-Aldrich), sulfuric acid (H_2_SO_4_, 10 Normal, Ricca Chemical Company), anhydrous dichloromethane (DCM, Sigma-Aldrich), N,N-dimethylformamide (DMF, HPLC grade, VWR), and lithium bromide (LiBr, anhydrous, 99.99%, VWR) were used without further purification.

### General Measurements

The redox potential change of the reaction system during polymerization was monitored by a Benchtop pH/mV Meter (Sper Scientific Direct) equipped with a MI-800 Micro-ORP Electrode (Microelectrodes Inc.), redox potential values were collected once per second. The hydrodynamic size of samples collected at different time points was measured by dynamic light scattering (DLS) analysis using a Delsa Nano C particle size and zeta potential analyzer (Beckman Coulter, Inc.). ^1^H-NMR spectra of monomer and polymers were recorded on a 500 MHz Varian Unity/Inova 500B spectrometer using methanol-*d*_4_ as the solvent. Morphology of the self-assembled structures was observed under transmission electron microscope (TEM, FEI Tecnai Cryo-Bio 200KV FEG TEM), field scanning electron microscope (FESEM, Supra 55VP) and/or Scanning electron cryomicroscopy (Cryo-SEM) [Zeiss NVision 40 focus ion beam scanning electron microscope (FIB-SEM)].

### Method

The BZ coupled PISA (BZ-PISA) process is briefly described here. 17.3 mg PEG-CTA (8 μmol) was dissolved in 15.465 mL ultrapure water. 1 mL malonic acid (MA, 0.6 M), 2 mL sodium bromate (NaBrO_3_, 1.0 M), 1.2 mL H_2_SO_4_ (5.0 M), 206 μL MEA monomers [1800 μmol, target degree of polymerization (DP_target_) = 200], and 0.235 mL Ru(bpy)_3_Cl_2_ (8.5 mM) were added in sequence and stirred at 200 rpm at room temperature. The redox potential change of the reaction system was monitored just after the addition of all reactants. An aluminum foil was applied to protect the reaction vessel from room light. At pre-set time points (0, 30, 60, 90, and 120 min), 60 μL reaction mixture was sampled out, neutralized with proper amount of NaOH, freeze-dried to remove unreacted monomer and then dissolved in 540 μL methanol-*d*_4_ for ^1^H-NMR measurement. Fresh samples collected at 30, 60, 90, and 120 min were used for DLS analysis immediately. The morphology of the self-assembled structures of samples collected at 30, 60, 90, and 120 min was observed by TEM, SEM and/or Cryo-SEM. After 2 h reaction, the reaction mixture was collected, neutralized and then dialyzed against deionized water using a dialysis tubing with a molecular cut-off of 1000 Da for 3 days. Water was changed every 2 h in the beginning 6 h and every the other day after.

BZ-PISA of PEG-b-PMEA with a DP_target_ = 400 and 600 were also conducted. The amount of monomer was kept unchanged, but the amount of PEG-CTA was adjusted accordingly to obtain a DP_target_ = 400 or 600. Pure BZ reaction was also conducted using the same recipe without adding PEG-CTA and monomer.

## Data Availability Statement

The raw data supporting the conclusions of this article will be made available by the authors, without undue reservation.

## Author Contributions

ZL drafted the manuscript. JG conducted the experiments.

## Conflict of Interest

The authors declare that the research was conducted in the absence of any commercial or financial relationships that could be construed as a potential conflict of interest.
