# Supplementary material for: Growing Polymer Vesicles Generated by Polymerization Induced Self-Assembly Coupled With a Living Chemical Reactor
Source: Front Bioeng Biotechnol. 2020 Sep 1;8:1018. doi: 10.3389/fbioe.2020.01018 (PMC7490293; doi:10.3389/fbioe.2020.01018)
Supplement: Supplementary file 1 [file Image_1.pdf]

Growing polymer vesicles generated by polymerization induced self-assembly coupled with a living chemical reactor

Zhihui Lu [1,*], Jinshan Guo[2]

[1]Department of Histology and Embryology, School of Basic Medical Sciences, Southern Medical University, Guangzhou, 510515, China.
*Corresponding author, Email: luzhihui107@sohu.com
[2]Department of Earth and Planetary Science and Origin of Life Initiative, Harvard University, 20 Oxford Street, Cambridge, Massachusetts, 02138, USA.

Key words:
Chemical oscillation; PISA; polymeric vesicles; self-assembly; biomimetics

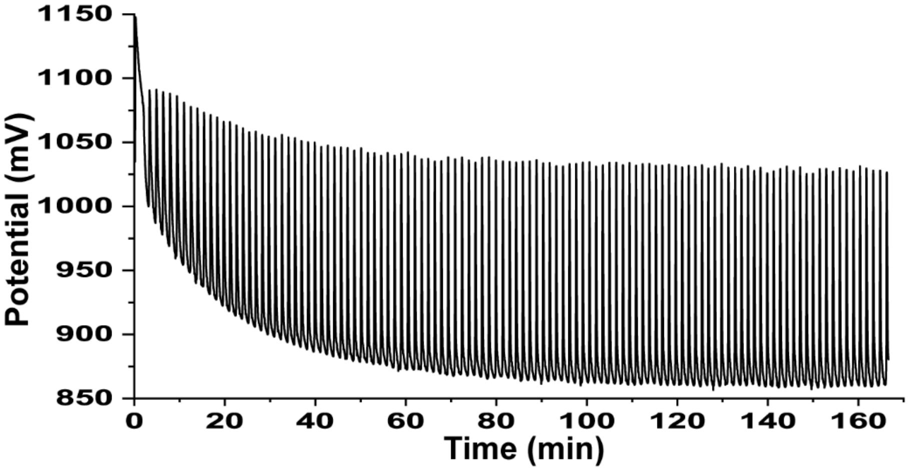

Figure S1. The redox potential oscillation curve in pure BZ oscillator.

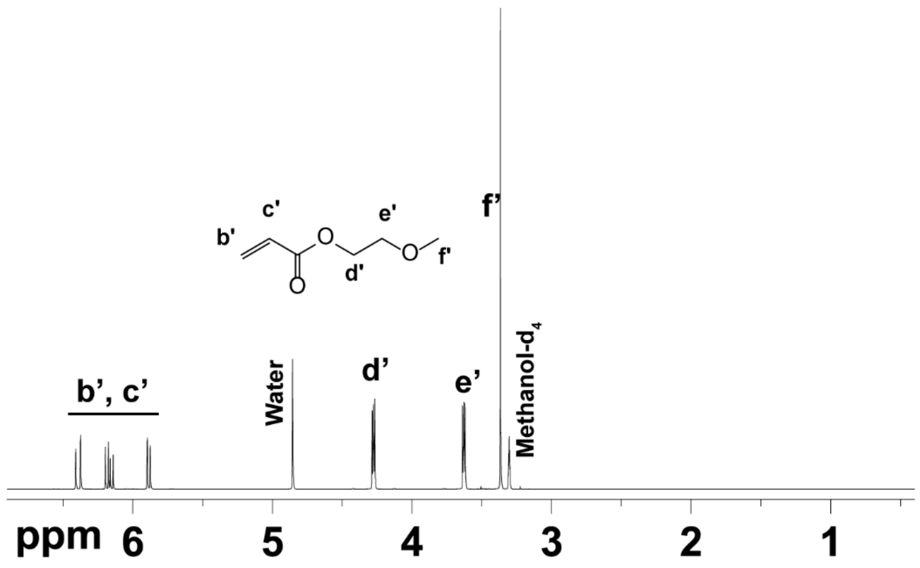

Figure S2. ^{1}H-NMR spectrum of 2-methoxyethyl acrylate (MEA) in methanol-d_4.

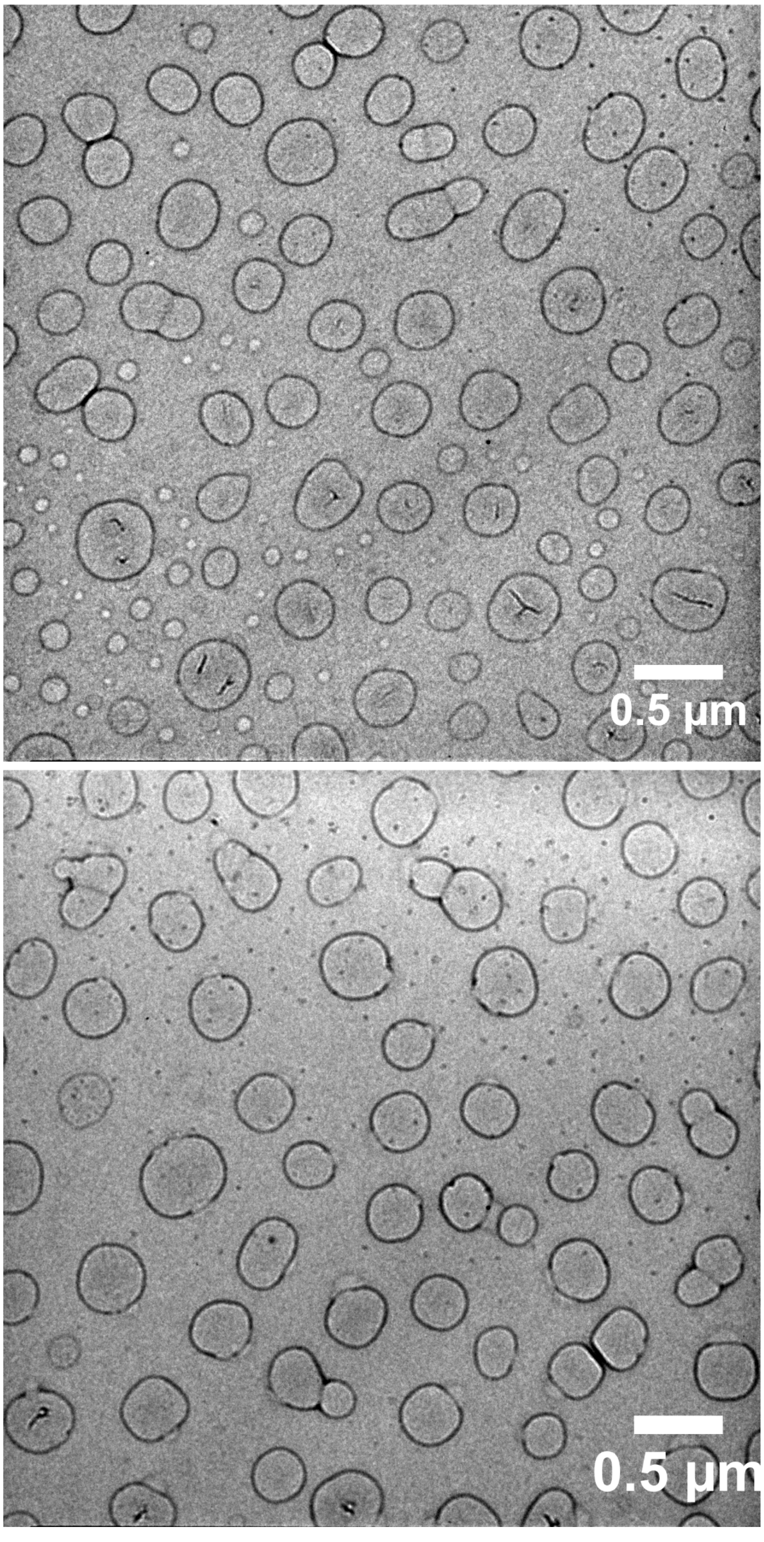

Figure S3. Representative TEM images of the BZ-PISA for PEG-b-PMEA$_{200}$ sample at 60 min.
